# Metabolomic profiling of amino acids study reveals a distinct diagnostic model for diabetic kidney disease

**DOI:** 10.1007/s00726-023-03330-0

**Published:** 2023-09-22

**Authors:** Jiao Wang, Chunyu Zhou, Qing Zhang, Zhangsuo Liu

**Affiliations:** 1 Department of Geriatric Endocrinology, The First Affiliated Hospital of Zhengzhou University, Zhengzhou, 450000 China; 2 Research Institute of Nephrology, Zhengzhou University, Zhengzhou, 450000 China; 3 Henan Province Research Center For Kidney Disease, Zhengzhou, 450000 China; 4 Blood Purification Center, The First Affiliated Hospital of Zhengzhou University, Zhengzhou, 450000 China; 5 Traditional Chinese Medicine Integrated Department of Nephrology, The First Affiliated Hospital of Zhengzhou University, Zhengzhou, 450000 China

**Keywords:** Diabetic kidney disease, Amino acids, Metabolomics, LC–MS/MS, Diagnostic model

## Abstract

Diabetic kidney disease (DKD), a highly prevalent complication of diabetes mellitus, is a major cause of mortality in patients. However, identifying circulatory markers to diagnose DKD requires a thorough understanding of the metabolic mechanisms of DKD. In this study, we performed ultra-performance liquid chromatography tandem mass spectrometry (UPLC-MS/MS) to reveal altered metabolic profiles of amino acids (AAs) in patients with DKD. We found decreased plasma levels of histidine and valine, increased urine levels of proline, decreased urine levels of histidine and valine, and increased saliva levels of arginine in patients with DKD compared with the levels in patients with type 2 diabetes mellitus (T2DM) and in healthy controls. Our analyses of the key metabolites and metabolic enzymes involved in histidine and valine metabolism indicated that the AAs level alterations may be due to enhanced carnosine hydrolysis, decreased degradation of homocarnosine and anserine, enhanced histidine methylation, and systemic enhancement of valine metabolism in patients with DKD. Notably, we generated a distinct diagnostic model with an AUC of 0.957 and an accuracy up to 92.2% on the basis of the AA profiles in plasma, urine and saliva differing in patients with DKD using logistic regression and receiver operating characteristic analyses. In conclusion, our results suggest that altered AA metabolic profiles are associated with the progression of DKD. Our DKD diagnostic model on the basis of AA levels in plasma, urine, and saliva may provide a theoretical basis for innovative strategies to diagnose DKD that may replace cumbersome kidney biopsies.

**Supplementary Information:**

The online version contains supplementary material available at 10.1007/s00726-023-03330-0.

## Introduction

Diabetic kidney disease (DKD), the single strongest predictor of mortality in patients with diabetes mellitus, is the leading cause of kidney failure worldwide (Mohandes et al. 2023). More than one-third of patients with diabetes mellitus develop DKD, and half of those eventually progress to end-stage renal disease (ESRD) (Park 2014). Despite this burden, the pathophysiological mechanism underlying DKD remains to be elucidated. The clinical DKD manifestations are heterogeneous, and no available biomarkers can accurately predict kidney function decline. In addition, considering that many patients with type 2 diabetes mellitus (T2DM) and DKD present a non-albuminuric phenotype, the eGFR appears to be the only diagnostic biomarker of DKD useful in the clinical practice (Agarwal et al. 2022). Thus, a clear understanding of DKD pathophysiological mechanisms is urgent to improve the status of patients with diabetes mellitus.

Recently, metabolomics is emerging as an attractive tool for biomarker discovery in diabetes and its complications. Both non-targeted metabolomics and targeted metabolomics (amino acids metabolism, lipids metabolism and nucleic acids metabolism) were reported to have the strong association with the progression of DKD. Among these involved metabolic pathways, the metabolism of amino acids (AAs) was coming into focus. For instance, Hirayama identified a panel of candidate markers of DKD and found between albumin‐to‐creatinine ratio and several other metabolites such aspartate, citrulline, symmetric dimethylarginine, and kynurenine suggesting DKD can be diagnosed through metabolome analysis (Hirayama et al. 2012). In a study with more than 1000 patients with DKD, Kwan developed a prognostic model combining metabolites such as 3‐hydroxyisobutyrate, an intermediate catabolite of valine, which reduced in the urine of DKD with other clinical variables managing to predict with great accuracy future decreases in eGFR and showing high concordance for the development of ESRD (Kwan et al. 2020).

AAs are important metabolic intermediates of different processes and they constitute the basic structural units of proteins (Li et al. 2022). AA profiles get significantly altered under different conditions, including diabetes mellitus (Verrey et al. 2009). In addition, regulating amino acid intake has been reported to delay the development of kidney injury and other metabolic complications in patients with T2DM (Zimmet and Alberti 2016). We elucidated similar dysregulation patterns in a previous study, in which the main alterations associated with DKD were decreased levels of histidine and valine in plasma, making these levels potential biomarkers for early diagnosis of DKD in patients with T2DM (Zhou et al. 2021). In addition, the AAs levels in urine are affected by those in plasma and other renal disease factors (Zimmet and Alberti 2016). In contrast with plasma, analyses of urine and saliva are advantageous, because they can be collected non-invasively, simply, repeatedly, and without the need for trained personnel. Endogenous metabolites in saliva originate from the systemic circulation (Ch et al. 2019), and the salivary concentrations of solutes are correlated partially with their plasma concentrations. Therefore, urine and saliva are promising diagnostic body fluids. However, the simultaneous collection of different types of biofluids for comparisons of their metabolomic profiles is rare.

In this study, firstly, we measured the concentrations of 20 AAs in plasma and elucidated the reprogramming of the metabolic pathway. Then, we analyzed the metabolites and metabolic enzymes of histidine and valine for whose levels altered in plasma significantly. Secondly, we quantitatively estimated the amount of 20 AAs in urine and saliva, and compared the correlated levels of AAs in plasma and urine or saliva. Finally, we established a diagnostic model on the basis of the AA profiles in plasma, urine, and saliva of patients with DKD, to distinguish patients with DKD from those with T2DM and healthy controls.

## Materials and methods

### Study design and participants

The Ethics Committee of the First Affiliated Hospital of Zhengzhou University approved this study (2022-KY-1408-001) and we conducted it conforming to the tenets of the Declaration of Helsinki 1964 and its later amendments. We obtained written and informed consents from all participants. We recruited patients with DKD (DKD group), patients with T2DM (T2DM group), and healthy controls (control group) from the Department of Nephrology, the Department of Endocrinology, and the Department of Physical Examination, respectively, at the First Affiliated Hospital of Zhengzhou University. The inclusion criteria for participants are present in our previously published article (Zhou et al. 2021). Table 1 lists the general characteristics of the patients in each group.

**Table 1:** General characteristics of the study population

	CON (*n* = 30)	T2DM (*n* = 30)	DKD (*n* = 30)	*P1*	*P2*
Age, year	57.5 ± 9.2	61.2 ± 9.5	62.1 ± 12.9	0.725	0.434
Male/female, *n*	16/14	19/11	18/12	0.253	0.073
Hb, g/L	145.8 ± 12.3	132.3 ± 14.4	97.6 ± 22.3	< 0.001	< 0.001
Alb, g/L	49.8 ± 3.2	42.3 ± 3.8	35.0 ± 8.6	< 0.001	< 0.001
Scr, μM	73.0 ± 13.5	69.6 ± 15.3	428.7 ± 304.8	< 0.001	< 0.001
TC, mM	4.3 ± 0.6	3.9 ± 0.9	5.0 ± 1.4	< 0.001	0.012
TG, mM	1.1 ± 0.5	1.7 ± 0.9	1.7 ± 0.9	0.984	< 0.001
LDL-C, mM	2.1 ± 0.8	2.4 ± 0.9	3.2 ± 1.7	0.011	0.001
HDL-C, mM	1.3 ± 0.4	1.1 ± 0.2	1.1 ± 0.4	0.405	0.015
PTH, pg/mL	NA	31.5 ± 15.5	119.9 ± 84.4	< 0.001	NA
HbA1c, %	NA	7.2 ± 1.4	9.0 ± 2.2	< 0.001	NA
UAER, mg/24h	NA	11.8 (2.0–38.0)	5190.0 (160.0–11550.0)	< 0.001	NA
eGFR, mL/min/1.73 m^2^	112.8 ± 14.9	98.2 ± 16.3	37.5 ± 34.8	< 0.001	< 0.001
Comorbidity					
CAD, *n*	0	17	20	0.430	< 0.001
HTN, *n*	0	11	21	0.010	< 0.001
DR, *n*	0	9	23	< 0.001	< 0.001
DPN, *n*	0	17	22	0.180	< 0.001

PTH HbAlc, and UAER values were not tested for healthy individuals in regular health examination

Continuous variables are presented as mean ± SD and were compared with the ne-way ANOVA test. Categorical variables were compared by Chi-square test or Fisher’s exact test and presented as counts. *P1*: DKD vs T2DM group; *P2*: DKD vs CON group

*Alb* albumin, *CAD* coronary artery disease; *CON* healthy controls; *DKD* diabetic kidney disease; *DPN* diabetic peripheral neuropathy; *DR* diabetic retinopathy; *eGFR* estimated glomerular filtration rate; *Hb* hemoglobin; *HbAlc* glycated hemoglobin; *HDL-C* high density lipoprotein cholesterol; *HTN* hypertension; *LDL-C* low density lipoprotein cholesterol; *NA* not available; *PTH* parathyroid hormone; *Scr* serum creatinine; *T2DM* type 2 diabetes mellitus; *TC* total cholesterol; *TG* triglyceride; *UAER* urinary albumin excretion rate

### Preparation samples for metabolomic profiling

We drew 2 mL intravenous blood samples from volunteers after overnight fasting under aseptic conditions and mixed the samples into anticoagulant tubes with ethylenediaminetetraacetic acid (EDTA). For the urine samples, participants filled approximately 2 mL of a sterile plastic bottle with a urine sample collected after 12 h of fasting in the early morning. We centrifuged the collected plasma and urine samples at 1500 rpm for 10 min at 4˚C, and we kept the supernatants at − 80 °C for further UPLC-MS/MS detection. After washing their mouths with 2 mL of saline, the participants collected saliva samples in the early morning after overnight fasting. The saliva samples were flash frozen in liquid nitrogen and then stored under – 80 °C until the experimental analysis.

We prepared stock solutions of AAs by dissolving the standard compounds in water or dimethylsulfoxide at concentrations ranging from 1 to 100 mM and stored them in brown volumetric flasks at − 80 ℃ until use. We diluted an isotope-labeled AA mix with acetonitrile (ACN) at 100 nM to use as an internal standard (IS). In addition, we prepared working solutions of the AAs by diluting stock solutions into seven different batches (10–1000 nM) with ACN. The plasma, urine, and saliva samples and standard curves were prepared using a protein precipitation extraction method.

### Metabolomic profiling of AAs in samples

We used the same AA detection protocol described in our previous published study (Zhou et al. 2021). Briefly, we detected AAs in samples using UPLC-MS/MS in positive ion multiple reaction monitoring mode on a UPLC-30ADvp series UPLC instrument (Shimadzu, Kyoto, Japan) with an SIL-30-AC autosampler, CTO-20AC column oven, and API 6500 triple-quadrupole source (Applied Biosystems Sciex, Toronto, ON, Canada). Supplemental Table 1 and 2 list the detailed UPLC-MS/MS conditions for AAs and isotope-labeled AAs. Supplemental Table 3 shows the gradient program for liquid chromatography. We used the Analyst v1.6.2 software for data acquisition and processing.

### Analysis of key metabolites and enzymes in plasma samples

We extracted total RNA samples from whole blood cells using the RNeasy Mini Kit (#74104; Qiagen, Valencia, CA, USA) and used them for reverse transcription to obtain cDNA using the RevertAid First Strand cDNA Synthesis Kit (K1622; Thermo Fisher Scientific, Waltham, MA, USA). We run quantitative real-time PCR reactions with TB Green Premix Ex Taq (#RR820; Takara Bio, Dalian, China). We used the glyceraldehyde 3-phosphate dehydrogenase (GAPDH) gene for target mRNA normalization. Supplemental Table 4 shows the primer sequences used. We measured plasma CNDP1 protein levels by enzyme-linked immunosorbent assays (#EH1356; FineTest, Wuhan, China) according to the manufacturer’s instructions.

We obtained relative quantities of key metabolites, including 1-methylhistidine (1-MH), 3-methylhistidine (3-MH), ergothioneine, carnosine, homocarnosine, anserine, trans-urocanate, and 3-methyl-2-oxobutyrate by UPLC-MS/MS (Supplemental Table 5). Supplemental Table 5 details the information for metabolites, and the Supplemental Materials and Methods describe the methodology. Supplemental Table 6 contains the liquid chromatography gradient program for key metabolites.

### Statistical analysis

We entered the data obtained from this study into a master chart to tabulate them. We calculated frequencies, percentages, means, standard deviations (SD), medians, and minimum and maximum values of variables. We applied one-way ANOVA and Kruskal Wallis tests for comparisons between different groups. In addition, we calculated the corresponding area under the ROC curve (AUC), cut-off values, sensitivity, specificity, and accuracy to evaluate the predictive performances of key AAs for DKD. We assessed correlations between plasma and urine or plasma and saliva variables by applying a Pearson correlation test. We considered *P* values < 0.05 as statistically significant. We conducted all data analyses using the Statistical Package for Social Sciences (SPSS) v21.0 (Armonk, NY, USA).

## Results

### Altered metabolism of 20 AAs in plasma of patients with DKD

We collected participant samples of blood, urine, and saliva for the control, T2DM, and DKD groups between October 2021 and September 2022. We obtained the same numbers of different samples from each individual. The plasma levels of 11 of the 20 AAs in the DKD group were significantly altered compared with those in the T2DM or control groups (*p* < 0.05). More specifically, we observed significantly reduced levels of histidine and valine in the plasma of patients with DKD when comparing them to the same levels in individuals of the T2DM or control groups (*p* < 0.05) (Supplemental Fig. 1 and Supplemental Table 7).

**Fig. 1:**
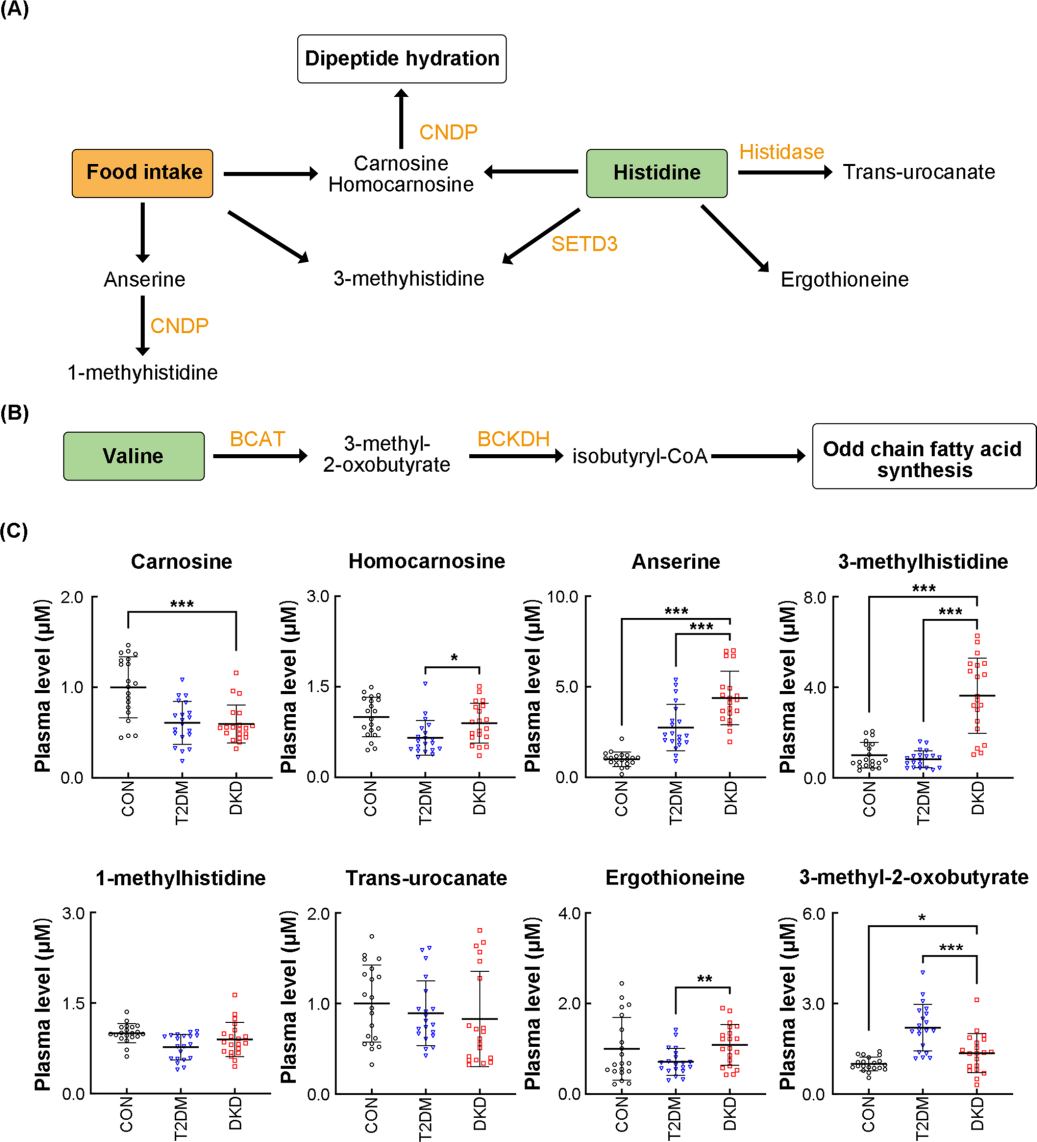
The metabolic pathways of histidine and valine are significantly different among DKD, T2DM and CON groups. Diagram of histidine metabolic pathway (**A**). Diagram of valine metabolic pathway (**B**). Circulating levels of metabolite of histidine and valine metabolism (**C**). *P < 0.05, **P < 0.01, ***P < 0.001

To examine the possible mechanisms underlying the observed changes in histidine metabolism in patients with DKD, we measured the relative levels of histidine metabolites in plasma from control, T2DM, and DKD groups (*n* = 20 per group) (Fig. 1A, C and Supplemental Table 8). Supplemental Fig. 2 shows the chromatogram of histidine and valine metabolites. The levels of carnosine, the major endogenous histidine-containing dipeptide (HDP), was significantly reduced both in T2DM and DKD groups compared to the levels in the control group (*p* < 0.001). The homocarnosine levels were reduced in the T2DM group compared to the levels in the control group (*p* < 0.001), but the same were increased in the DKD group compared to the levels in the T2DM group (*p* < 0.001). In contrast, anserine levels were significantly increased in both T2DM and DKD groups, when compared to the levels in the control group (*p* < 0.001), and they were higher in the DKD than in the T2DM group (*p* < 0.001). The levels of 3-MH, a methyl-containing derivative of histidine, were significantly higher (*p* < 0.001) in the DKD group than in the others, but the 1-MH levels were similar in the DKD and T2DM groups. The levels of trans-urocanate (a deamination product of histidine) and ergothioneine (a sulfur-containing derivative of histidine) were similar in all the groups.

**Fig. 2:**
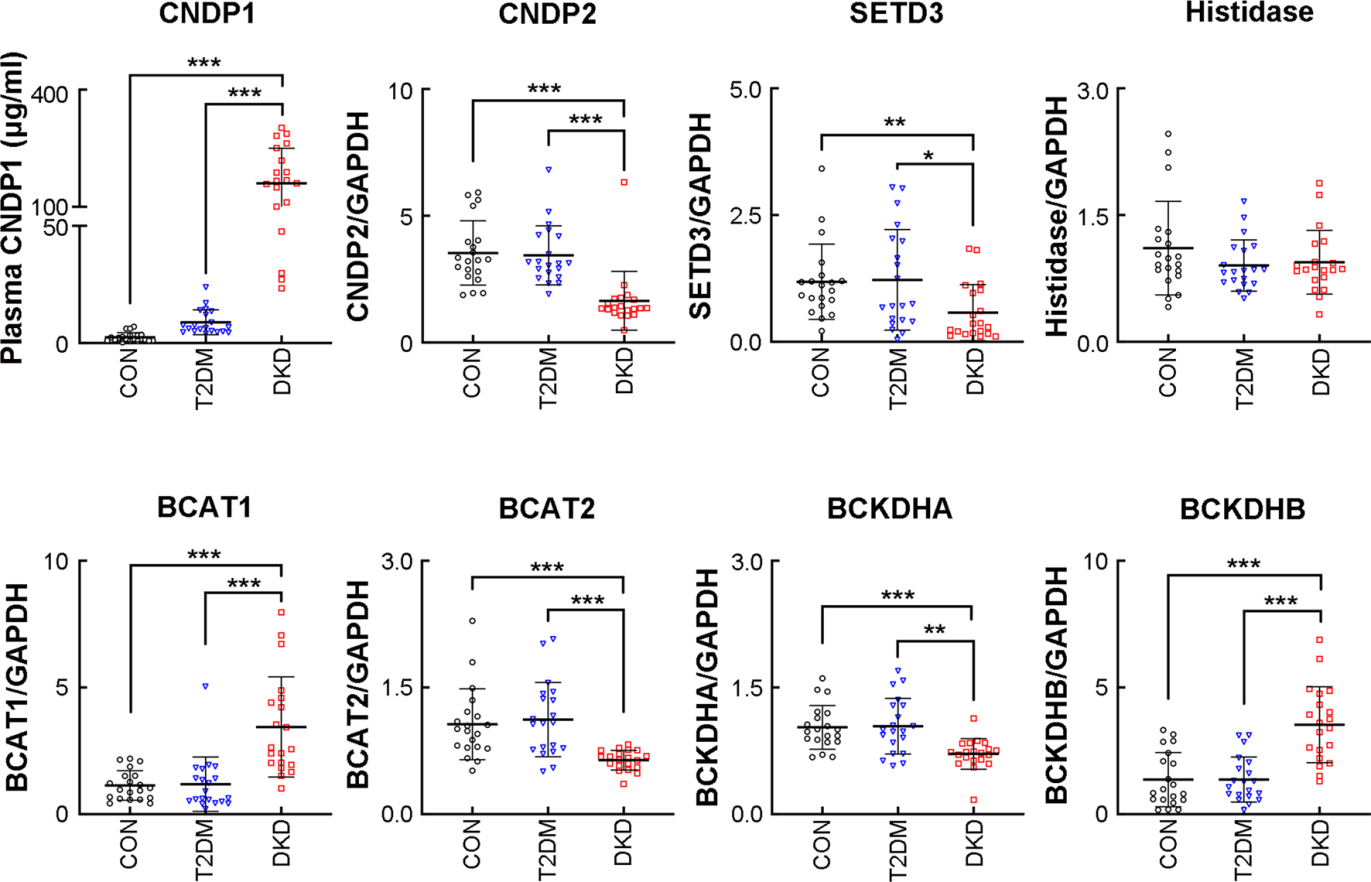
Circulating levels of major enzymes of histidine and valine metabolism. CNDP1 was detected by an enzyme-linked immunosorbent assay in plasma, while CNDP2, SETD3, Histidase, BCAT1, BCAT2, BCKDHA, and BCKDHB mRNA expression levels were detected using quantitative real-time PCR of whole blood cells. CNDP1, carnosinase 1; CNDP2, carnosinase 2; BCAT1, branched-chain aminotransferase 1; BCAT2, branched-chain aminotransferase 2; BCKDHA, branched-chain ketoacid dehydrogenase A; BCKDHB, branched-chain ketoacid dehydrogenase B; SETD3, SET domain-containing 3. *P < 0.05, **P < 0.01, ***P < 0.001

To confirm the observed changes, we assessed the transcript levels of enzymes involved in histidine metabolism in the circulation (Fig. 2). In the case of carnosine, homocarnosine and anserine metabolism, the plasma levels of CNDP1 were significantly higher (*p* < 0.001), whereas those of carnosine dipeptidase 2 (CNDP2) were lower (*p* < 0.001) in the DKD group than in the T2DM and control groups. The same decrease was also present for the level of SET domain-containing 3 (SETD3), a histidine-methyl-transferase. In contrast, the circulating mRNA level of histidase, which regulates trans-urocanate and ergothioneine metabolism, was not significantly altered in the DKD group. These results suggest that patients with DKD present enhanced carnosine hydrolysis, decreased degradation of homocarnosine and anserine, as well as enhanced histidine methylation in the circulating blood.

We also measured metabolites and corresponding metabolic enzymes of valine metabolism (Fig. 1B, C and Supplemental Table 8). The transcript levels of 2 circulating branched-chain aminotransferases (BCATs) showed opposite trends: in the DKD group, BCAT1 was upregulated (*p* < 0.001) whereas BCAT2 was downregulated compared to the levels in the T2DM and control groups. 3-methyl-2-oxobutyrate a product of BCAT was significantly high (*p* < 0.001) in patients with T2DM. Moreover, the mRNA levels of the downstream enzyme branched-chain ketoacid dehydrogenase A (BCKDHA) were lower (*p* < 0.01), whereas those of BCKDHB were higher (*p* < 0.001) in the DKD group than in the T2DM and control groups. These results confirm that valine metabolism is significantly altered in the circulating blood of patients with DKD.

### The distinct metabolic profile of 20 AAs in urine and saliva in patients with DKD

The urine levels of 13 of the 20 AAs in the DKD group were significantly altered compared with those in the T2DM or control groups (*p* < 0.05) (Supplemental Table 9). In addition, the concentrations in urine of histidine, valine, and proline differed between both DKD and T2DM groups and between DKD and control groups (Supplemental Table 9). Among the above three AAs, we observed a significant reduction in the levels of histidine and valine (*p* < 0.05) and an increase in the levels of proline (*p* < 0.05) in the DKD group (Supplemental Fig. 1). Meanwhile, in saliva, only the levels of arginine presented a significant alteration, the levels were higher in the DKD group than in others (*p* < 0.05) (Supplemental Fig. 1 and Supplemental Table 10).

**Fig. 3:**
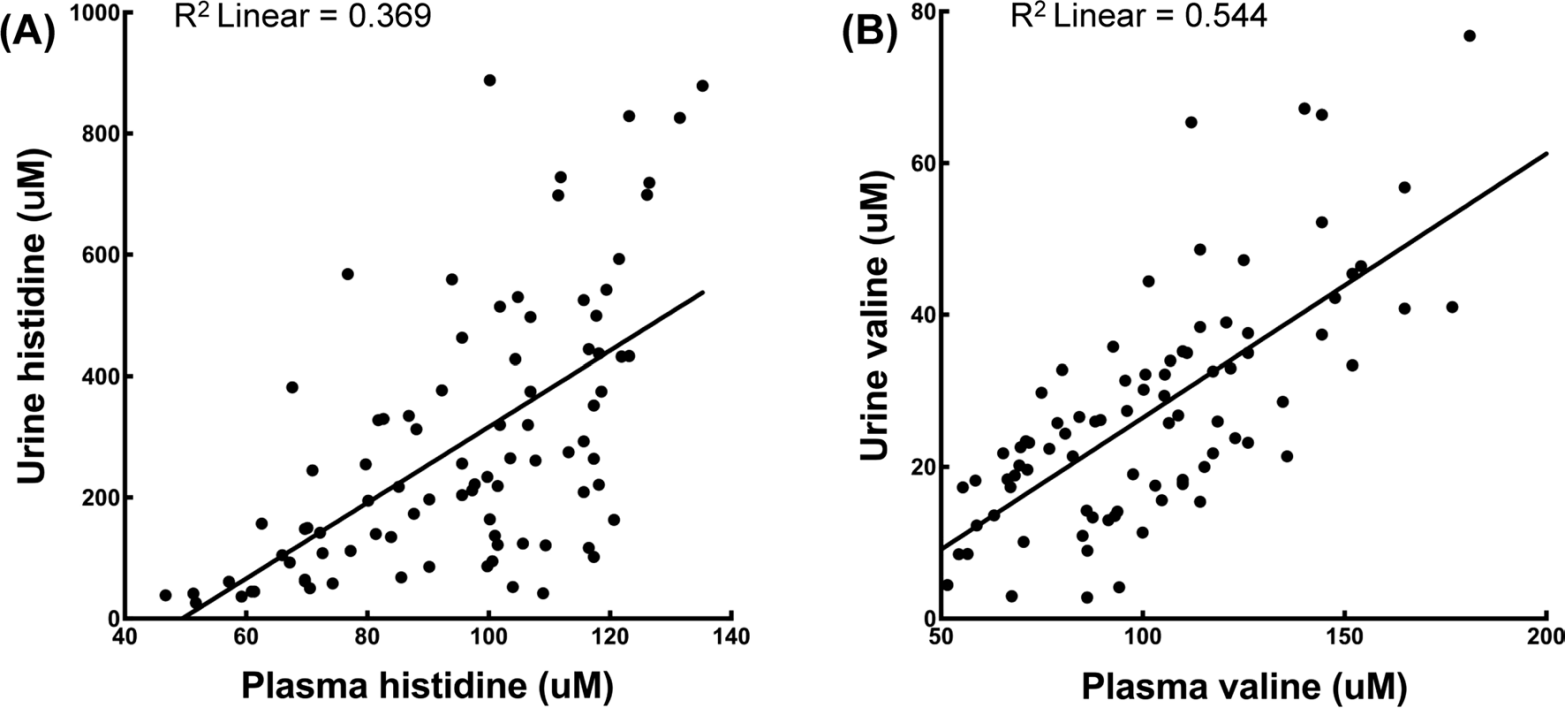
Association between the amino acids concentrations in the plasma and urine. Correlation is significant at the 0.0001 level (2-tailed). Pearson’s Correlations Coefficient: 0.538

Next, we conducted a Pearson correlation analysis to visualize any associations between the levels of amino acids in plasma and either urine or saliva. We found a strong positive correlation (0.6 < *r* < 0.8, *p* < 0.05) in the levels of valine, as well as a moderate correlation (0.4 < *r* < 0.6, *p* < 0.05) in the levels of histidine between plasma and urine (Fig. 3 and Supplemental Table 11).

### Validation of the diagnostic model

**Fig. 4:**
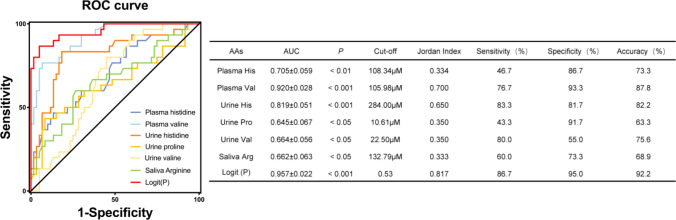
ROC curve and AUC for different model. Plasma histidine (6C89C0 line), plasma valine (A5CEE9 line), urine histidine (FD8008 line), urine proline (FFC000 line), urine valine (FFE38B line), saliva arginine (92D050 line), Logit(P) (FF0000 line). Accuracy = (*A* × sensitivity + *B* × specificity)/(*A* + *B*), where *A* is the number of participants in the corresponding disease group and *B* is the number of participants in the corresponding control group

To distinguish participants with DKD from those with T2DM and healthy controls, we used logistic regression and established a diagnostic model based on the levels of AAs that differed in the DKD group from those in the T2DM and control groups in plasma, urine, and saliva. The diagnostic regression equation was as follows: Logit (P) = 10.673–0.026 × log (plasma histidine)-0.099 × log (plasma valine)-0.004 × log (urine histidine) + 0.002 × log (urine valine) + 0.064 × log (urine proline) + 0.006 × log (saliva arginine). Our results revealed that the corresponding ROC curve had an AUC of 0.957 (95% CI 0.915–1.000) with a Youden index *J* of 0.53 (Fig. 4 and Supplemental Table 12), indicating an excellent predictive performance for the diagnostic model. To validate our results, we analyzed the association of the samples in the validation datasets. Only eight samples in the validation set had uncertain classifications, and the accuracy of the model was 92.2% (Fig. 4). The AUC of the combined diagnostic model was better than that for each individual AA, indicating that the diagnostic equation based on these six AAs yielded the highest AUC value and should significantly improve the diagnostic performance of the model for patients with DKD.

## Discussion

In this study, we performed a UPLC-MS/MS-based metabolomics analysis to reveal the metabolic profile of 20 AAs in plasma, urine, and saliva samples of patients with DKD. After that, to predict the presence of DKD, we established a distinct diagnostic model based on six differential AAs including plasma histidine and valine; urine histidine, valine, and proline; and, saliva arginine with high accuracy, and we confirmed its performance using the validation set (92.2% by diagnostic feature).

In our study, we found decreased plasma levels of histidine and valine in patients with DKD, these results are consistent with our previous findings (Zhou et al. 2021). Further exploration of the key metabolites and enzymes of histidine metabolism indicated enhanced carnosine hydrolysis, decreased degradation of homocarnosine and anserine, and increased histidine methylation in the circulating blood of patients with DKD. Histidine exerts anti-inflammatory and antioxidant effects by a combination of free radical elimination and metal chelation (Babizhayev 2006). Insufficient plasma histidine levels are associated with persistent inflammation and oxidative stress in renal diseases, which can be relieved by dietary histidine supplementation (Lee et al. 2005; Watanabe et al. 2008), and the abnormal levels are probably caused by histidine metabolism abnormalities (Lee et al. 2005). Carnosine is an important HDP with renoprotective effects in patients with T2DM and DKD. In rodent models of T2DM, oral carnosine supplementation alleviates structural and functional renal damage via anti-inflammatory, antioxidant, anti-glycation, and reactive carbonyl-quenching mechanisms (Holeček 2020). Results of a clinical trial showed that carnosine supplementation over 12 weeks improved glucose tolerance (Albrecht et al. 2017; de Courten et al. 2016). Our results are consistent with those of another study indicating that overexpression of CNDP1, the metabolic enzyme of carnosine, is associated with a risk for poor diabetes control (and a high risk for T2DM and DKD). CNDP2 is another carnosinase that mainly hydrolyzes other HDP substrates (homocarnosine and anserine) under nonphysiological conditions (Boldyrev et al. 2013). In our study, homocarnosine was elevated in patients with DKD due to attenuated activation of the downstream enzyme CNDP2; homocarnosine is endogenously synthesized in skeletal muscle from histidine and γ-aminobutyric acid and its concentration is barely influenced by a low dietary protein intake.

Little is known about the function of the methylated form of carnosine. Anserine is the exogenous HDP synthesized through carnosine methylation in most mammals, fish, and amphibians but not in humans (Derave et al. 2019). By exerting the same effect as carnosine, anserine may improve glucose metabolism, proteinuria, and vascular permeability under diabetic conditions due to its resulting glycation inhibition, oxidative damage reduction, and antioxidant activity enhancement (Derave et al. 2019). Our data show that the plasma levels of anserine were much higher in patients with DKD than in other participants; this may be attributable to a decrease in anserine degradation due to the low expression of CNDP2 (Peters et al. 2011). 1-MH is derived from the degradation of exogenous anserine (which is present in the meat of most fish, but not in humans) and is a biomarker of protein intake (Stifel and Herman 1971). In contrast, 3-MH is endogenously synthesized only in muscle by the methylation histidine residues and it gets excreted in the urine without being metabolized (Holeček 2020). We found that 3-MH was increased in participants of the DKD group, whereas 1-MH was unchanged in all participants. The 3-MH increases are likely due to higher muscle protein turnover or sarcopenia rather than to restricted dietary protein intake (Kim et al. 2010).

Valine metabolism was also altered in patients with DKD. Valine is transaminated by BCAT in mitochondria to generate 3-methyl-2-oxobutyrate, and then it gets dehydrolyzed by the BCKDH complex, which produces isobutyryl-coenzyme A, a raw material for odd chain fatty acid (OCFA) synthesis. Valine contributes to the increased levels of liver and circulating OCFA in patients with T2DM via 2 distinct mechanisms–increased α-oxidation and de novo OCFA lipogenesis (Bishop et al. 2020). We observed an increase in 3-methyl-2-oxobutyrate in patients with T2DM, but the circulating levels of BCAT and BCKDH, the enzymes responsible for its metabolism, were not significantly altered. This may be because valine is mainly metabolized in the mitochondria of skeletal muscle and the levels are lower in other peripheral tissues and in the circulation. In our study, we found that patients with DKD had lower plasma levels of 3-methyl-2-oxobutyrate and abnormal upregulation of circulating BCAT1 and BCKDHB, which can promote valine metabolism (Nie et al. 2018). Additional studies are needed to determine how the association between valine metabolism and a restricted dietary intake contributes to DKD development.

We observed lower levels of histidine and valine, as well as higher levels of proline in urine of patients with DKD than those in patients with T2DM or healthy controls. Hyperglycemia is considered the principal cause of the significantly altered urinary AA excretion pattern of patients with T2DM (Verrey et al. 2009). Bingham reported the prevalence of glycosuria-related AAs in all types of diabetes (Bingham et al. 2001). Bidi found that urine levels of aromatic amino acids, such as phenylalanine and tryptophan, sulfur-containing amino acids including cysteine, and basic amino acids, such as arginine, were significantly higher in patients with T2DM than in healthy controls (Bidi et al. 2020). However, the published data on urine amino acid patterns in DKD is scarce. Kim NH reported that the levels of methionine, valine, and leucine in urine increased in 8-week-old DKD mice, but then decreased to normal healthy control levels at 20 weeks (Kim et al. 2018). Bingham found that the levels of urinary AAs were high in T2DM regardless of the presence of chronic renal failure. Urinary AA levels reflect alterations in blood AAs levels (Babizhayev et al. 1994). Our findings revealed that the urine levels of histidine and valine were significantly lower in patients with DKD than those in patients with T2DM and healthy controls, a fact that may be due to the reduction of histidine and valine in plasma. Indeed, we found the levels of histidine and valine in urine to be moderately to strongly correlated with the levels in plasma.

The proline plasma levels have been shown to be increased in patients with T2DM, obesity, and insulin resistance; such persistent alterations lead ultimately to impaired insulin secretion, systemic glucose homeostasis disruption, and other dysfunctions (Liu et al. 2016). The localized levels of proline get increased due to excessive redox reactions under chronic inflammation and they lead to fibrotic tissue remodeling (Distler et al. 2019). Proline is indispensable in local tissues for collagen synthesis, but large accumulations result in maladapted tissue architectures (Tarbit et al. 2019; Vettore et al. 2021). The increased urinary excretion and decreased plasma levels of proline in patients with DKD in our study are probably due to the local inflammation and fibrosis in the kidney. However, the mechanisms leading to increased AA excretion are unclear. Glucose may also cause decrease AA reabsorption in the kidneys by depolarizing the electrical gradient of sodium-dependent amino acid transporters in the proximal renal tubules.

We found the saliva levels of most AAs in patients with T2DM and DKD to be higher than those in healthy controls. However, most studies on the levels of AAs in saliva have been conducted on animals (Distler et al. 2019). The AA contents in saliva may inadequately reflect those in serum, because the components of saliva are passively diffused from serum and also actively transported through the oral mucosa or gums (Gross et al. 2005; Rheinberger and Böger 2014). We found the saliva levels of arginine in the DKD group were higher than in T2DM and healthy control groups. However, after analyzing possible correlations of the arginine levels between saliva and plasma, we found none. The mechanism causing the differing arginine levels in the DKD group remain to be elucidated.

To validate the diagnostic performance for AAs differing in the DKD group when compared to those in the other groups in plasma, urine, and saliva, we conducted a logistic regression analysis to generate an optimal diagnostic model. The AUC of our diagnostic model was 0.957 in the corresponding ROC curve, with high specificity and sensitivity. The glomerular filtration rate (eGFR) is the gold standard for evaluating renal function (Gross et al. 2005; Rheinberger and Böger 2014), but abnormalities are usually undetectable before the occurrence of substantial kidney injury in patients with DKD. DKD is usually diagnosed on the basis of histological features after an invasive puncture biopsy that poses risks of hematuria or perirenal hematoma (Stiles et al. 2000). The lack of suitable early detection methods is a major obstacle to the prevention and treatment of DKD. For our study, we applied targeted metabolomics to plasma, urine, and saliva AA levels, and we found the altered metabolic profiles of 20 AAs in patients with DKD. After screening the AAs with significant difference levels in different biofluids, we were able to establish a simple and accurate DKD diagnostic model.

Some limitations to our study need to be considered. First, the samples of non-albuminuric phenotype of DKD were very rare, therefore, we could not sufficiently determined the profiles of the 20 AAs at different stages of DKD in biofluids. Second, since saliva secretion was affected by oral related problems, such as dental caries, oral inflammation or infection and smoking, etc. We could exclude samples with visible blood contamination, but did not screening for other oral diseases of the participants. Third, as this was a cross-sectional study, the causal relationship between T2DM, DKD, and AA alterations should be further explored. Therefore, further investigation, including expanding the sample size and validating the diagnostic model, is imminent.

In conclusion, we explored here the metabolomic profiles of 20 AAs of plasma, urine, and saliva from patients with DKD. Logistic regression revealed a distinct diagnostic equation based on six differential AAs. In our study, the UPLC-MS/MS method was used for the first time to conduct metabolomic profiling of 20 AAs in human saliva of DKD. It was found that the elevated arginine level was the characteristic of the AAs in saliva of DKD patients, opening a new chapter for the study of the mechanism of DKD. In addition, our findings provided a theoretical basis regarding AAs as the non-invasive, real-time, fast and simple biomarker for the diagnosis and prediction of T2DM and DKD.

### Supplementary Information

Below is the link to the electronic supplementary material.

Supplementary file1 (DOCX 582 KB)

## Data Availability

The datasets generated and analysed during the current study are available from the corresponding author on reasonable request.
