# Supplementary material for: Metabolomic profiling of amino acids study reveals a distinct diagnostic model for diabetic kidney disease
Source: Amino Acids. 2023 Sep 22;55(11):1563–72. doi: 10.1007/s00726-023-03330-0 (PMC10689543; doi:10.1007/s00726-023-03330-0)
Supplement: Supplementary file 1 — Supplementary file1 (DOCX 582 KB) [file 726_2023_3330_MOESM1_ESM.docx]

Supplementary Material

Metabolomic profiling of amino acids study reveals a distinct diagnostic model for Diabetic kidney disease

Jiao Wang *, Chunyu Zhou, Qing Zhang, Zhangsuo Liu

*** Correspondence:** Dr. Qing Zhang: aquamarine61@163.com

Dr.Zhangsuo Liu: zhangsuoliu@zzu.edu.cn. Tel.: +86-0371-66271018

# Supplementary Data

*Recruitment criteria*

The detailed inclusion criteria for patients with type 2 diabetes mellitus (T2DM)-induced diabetic kidney disease (DKD group) were the following: 1) age between 18 and 75 years; 2) DKD diagnosis established at our hospital following the American Diabetes Association (ADA) criteria [1]; 3) absence of any other clinically diagnosed severe disease including but not limited to cancer; nervous, digestive, and mental disorders; or infectious diseases; and, 4) willingness to participate in the study.

The inclusion criteria for T2DM patients were the following: 1) age between 18 and 75 years; 2) T2DM diagnosis established at our hospital following ADA criteria [2]; 3) absence of albuminuria and/or reduced estimated glomerular filtration rate and without signs or symptoms of other primary causes of kidney damage; 4) absence of other clinically diagnosed severe disease including but not limited to cancer; nervous, digestive, and mental disorders; and infectious diseases; and 5) willingness to participate in the study.

The healthy volunteers were randomly selected from the Department of Physical Examination of our hospital and we sex- and age-matched them to the DKD and T2DM groups at a statistical significance level >0.05. The inclusion criteria for healthy volunteers were the following: 1) age between 18 and 75 years; 2) absence of self-reported or clinical diagnostic history of any chronic severe disease, including but not limited to cancer; nervous, renal, digestive, and mental disorders or infectious diseases within 3 months; 3) not receiving any medical treatment in the 3 months prior to blood collection; and 4) willingness to participate in the study.

*Reagents*

The standard compounds of 20 amino acids (AAs) were purchased from MedChem Express (Monmouth Junction, NJ, USA); the corresponding isotope-labeled AA mix was purchased from Sigma-Aldrich (St. Louis, MO, USA; #909653). Details of these standards are listed in Supplemental Tables 1 and 2. Acetonitrile (ACN, #A998-4), methanol (MeOH, #A452-4), and water (#34877) were purchased from Thermo Fisher Scientific (Waltham, MA, USA); 2-mL capped vials (#5183-4331) were from Agilent Technologies (Santa Clara, CA, USA); 1-methylhistidine (1-MH) (#67520; Sigma-Aldrich), 3-methylhistidine (3-MH) (#M9005; Sigma-Aldrich), anserine (#B24424; Yuanye Bio-Technology Co., Shanghai, China), carnosine (#HY-W013494; MedChem Express), ergothioneine (#HY-N1914; MedChem Express), homocarnosine (S182442; Sage Chemical, Hangzhou, China), trans-urocanate (SU8120; Solarbio Science & Technology, Beijing, China), and 3-methyl-2-oxobutyrate (#169975; Aladdin, Shanghai, China) were purchased from local distributors.

*Preparation of plasma, urine and saliva samples for metabolic profiling*

For sample preparation, we transferred 50 μL of thawed plasma, urine or saliva and 50 μL of ACN to a 1.5-mL tube containing 150 μL of internal standard (IS) (100 nM), and standard curves were generated by mixing 50 µL of plasma (mixed from 30 healthy individuals), 50 µL of working solution, and 150 µL of IS. We mixed all samples by vortexing for 10 min, followed by centrifugation at 4℃ and 15,000 rpm. The supernatants were transferred into 250-µL inserts fixed in 2-mL vials for further metabolic analyses.

*UPLC-MS/MS parameters*

[M+H]^+^ precursor ions were used for AAs and ISs. The optimized MS parameters of compounds were the following: capillary voltage, 5500 V; source temperature, 550°C; curtain gas (N_2_), 30 psi; collision gas, 10 psi; pressure for nebulization gas, 40 psi; evaporization gas; 40 psi; entrance potential, 50 V; and, collision cell exit, 10 V. The Tables S1 and S2 list the detailed declustering potential and collision energy as well as the precursor and dominant daughter ions of AAs and ISs.

Chromatographic separation was performed at 40℃ using an UPLC BEH amide column (2.1×100 mm, 3-μm particle size; Waters, Milford, MA, USA) equipped with a 1.7-μm VanGuard precolumn; the isocratic gradient elution program (Table S3) was run with mobile phase B (ACN) and mobile phase A (water containing 0.1% formic acid and 0.05% trifluoroacetic acid).

*Relative quantification of histidine and valine metabolites in plasma*

The stock solutions for 1-MH, 3-MH, anserine, carnosine, ergothioneine, homocarnosine, trans-urocanate, and 3-methyl-2-oxobutyrate were formulated by dissolving the standard compounds in water at concentrations of 100 mM and diluting them to 1 μM for MS analysis. The plasma concentration of these compounds was detected by UPLC-MS/MS in positive ion multiple reaction monitoring mode as described. Tables S5 and S6 list the MS parameters and gradient program for liquid chromatography.

# Supplementary Tables and Figures

## Supplementary Tables

**Supplemental Table 1.** MS parameters, measurement range, and quantification of 20 amino acids

| **Amino acid** | **MS number*** | **Abbreviation** | **ID** | **CAS number** | **Catalog ID^#^** | **MRM transition** | **DP (V)** | **CE (V)** | **Range (nM)** | **Linearity (R^2^)** |
| --- | --- | --- | --- | --- | --- | --- | --- | --- | --- | --- |
| Glycine | a | Gly | HMDB0000123 | 56-40-6 | HY-N0390 | 76→30 | 20 | 18 | 10-1000 | 0.9985 |
| L-Alanine | b | Ala | HMDB0000161 | 56-41-7 | HY-N0229 | 90→44 | 23 | 20 | 10-1000 | 0.9999 |
| L-Arginine | m | Arg | HMDB0000517 | 74-79-3 | HY-N0455 | 175→70 | 38 | 23 | 50-1000 | 0.9906 |
| L-Asparagine | c | Asn | HMDB0000168 | 70-47-3 | HY-N0667 | 133→116 | 35 | 13 | 10-1000 | 0.9951 |
| L-Aspartic acid | d | Asp | HMDB0000191 | 56-84-8 | HY-N0666 | 134→74 | 40 | 17 | 10-1000 | 0.9973 |
| L-Cysteine | e | Cys | HMDB0000574 | 52-90-4 | HY-Y0337 | 122→76 | 30 | 17 | 10-1000 | 0.9981 |
| L-Glutamic acid | f | Glu | HMDB0000148 | 56-86-0 | HY-14608 | 148→102 | 85.7 | 15 | 50-1000 | 0.9975 |
| L-Histidine | n | His | HMDB0000177 | 71-00-1 | HY-N0832 | 156→110 | 30 | 20 | 50-1000 | 0.9941 |
| L-Isoleucine | o | Ile | HMDB0000172 | 73-32-5 | HY-N0771 | 132→86 | 30 | 14 | 10-1000 | 0.9901 |
| L-Leucine | g | Leu | HMDB0000687 | 61-90-5 | HY-N0486 | 132→30 | 40 | 20 | 20-1000 | 0.9912 |
| L-Lysine | p | Lys | HMDB0000182 | 56-87-1 | HY-N0469 | 147→84 | 30 | 23 | 50-1000 | 0.9965 |
| L-Methionine | q | Met | HMDB0000696 | 63-68-3 | HY-N0326 | 150→133 | 24 | 12 | 10-1000 | 0.9978 |
| L-Ornithine | h | Orn | HMDB0000214 | 70-26-8 | HY-B1352 | 133.1→70.3 | 41 | 20 | 10-1000 | 0.9959 |
| L-Phenylalanine | r | Phe | HMDB0000159 | 63-91-2 | HY-N0215 | 166→120 | 40 | 20 | 10-1000 | 0.9977 |
| L-Proline | s | Pro | HMDB0000162 | 147-85-3 | HY-Y0252 | 116→70 | 60 | 27 | 10-1000 | 0.9933 |
| L-Serine | i | Ser | HMDB0000187 | 56-45-1 | HY-N0650 | 106→60 | 27 | 15 | 10-1000 | 0.9906 |
| L-Threonine | j | Thr | HMDB0000167 | 72-19-5 | HY-N0658 | 120→74 | 30 | 14 | 10-1000 | 0.9981 |
| L-Tryptophan | k | Trp | HMDB0000929 | 73-22-3 | HY-N0623 | 205→146 | 23 | 23 | 20-1000 | 0.9935 |
| L-Tyrosine | l | Tyr | HMDB0000158 | 60-18-4 | HY-N0473 | 182→136 | 36 | 17 | 10-1000 | 0.9964 |
| L-Valine | t | Val | HMDB0000883 | 72-18-4 | HY-N0717 | 118→72 | 40 | 13 | 10-1000 | 0.9964 |

*Peak number corresponding to the chromatogram shown in Figure 1A–D.

^#^Catalog ID corresponding to amino acids purchased from MedChem Express (Monmouth Junction, NJ, USA).

AA, amino acid; CE, collision energy; DP, declustering potential; MRM, multiple reaction monitoring; MS, mass spectrometry; nM, nanomole.

**Supplemental Table 2.** MS parameters of 20 isotope-labeled amino acids used as internal standards

| **Chemical** | **MRM transition** | **DP (V)** | **CE (V)** |
| --- | --- | --- | --- |
| Glycine-^13^C_2_,^15^N | 79→32.1 | 40 | 20 |
| L-Alanine-^13^C_3_,^15^N | 94.1→47.1 | 40 | 30 |
| L-Arginine-^13^C_6_,^15^N_4_ | 185.1→75.2 | 40 | 30 |
| L-Asparagine-^15^N_2_ | 135.1→75 | 80 | 30 |
| L-Aspartic acid-^13^C_4_,^15^N | 139.1→77 | 30 | 30 |
| L-Cystine-3,3,3′,3′-d_4_ | 245.2→122.2 | 30 | 25 |
| L-Glutamic acid-^13^C_5_,^15^N | 154→89.1 | 30 | 25 |
| L-Histidine-^15^N_3_ | 159→113 | 30 | 20 |
| L-Isoleucine-^13^C_6_,^15^N | 139→92.1 | 60 | 30 |
| L-Leucine-^13^C_6_,^15^N | 138.1→91 | 30 | 20 |
| L-Lysine-^13^C_6_,^15^N_2_ | 155.1→90.1 | 40 | 25 |
| L-Methionine-^15^N | 156→109.1 | 65 | 20 |
| L-Glutamine-^15^N_2_ | 149→131 | 40 | 15 |
| L-Phenylalanine-^13^C_9_,^15^N | 176.1→129 | 40 | 20 |
| L-Proline-^13^C_5_,^15^N | 122.1→75.1 | 30 | 20 |
| L-Serine-^13^C_3_,^15^N | 110.1→63 | 40 | 20 |
| L-Threonine-^13^C_4_,^15^N | 125.1→78.1 | 20 | 20 |
| L-Tryptophan-^15^N_2_ | 207.1→189.1 | 50 | 15 |
| L-Tyroxine-^13^C_9_,^15^N | 192.1→130.1 | 50 | 25 |
| L-Valine-^13^C_5_,^15^N | 124.1→77.1 | 25 | 20 |

CE, collision energy; DP, declustering potential; MRM, multiple reaction monitoring; MS, mass spectrometry.

**Supplemental Table 3.** Gradient program for liquid chromatography

| **Total time (min)** | **Flow rate (μl/min)** | **A^a^ (v/v, %)** | **B^b^ (v/v, %)** |
| --- | --- | --- | --- |
| 0.00 | 300 | 99.0 | 1 |
| 1.50 | 300 | 98.0 | 2 |
| 2.50 | 300 | 80.0 | 20.0 |
| 3.00 | 300 | 5.0 | 95.0 |
| 4.50 | 300 | 5.0 | 95.0 |
| 5.00 | 300 | 99.0 | 1.0 |
| 8.00 | 300 | 99.0 | 1.0 |

^a^Solvent A, water containing 0.1% formic acid (v/v) and 0.05% trifluoroacetic acid; ^b^Solvent B, acetonitrile.

**Supplemental Table 4.** Primer sequences for real-time quantitative PCR

| **Gene** | **Species** | **Forward** | **Reverse** |
| --- | --- | --- | --- |
| BCAT1 | Human | 5’-GGATAGAATGTATCGCTCTGCT-3’ | 5’-AGGCTCAGTTCCAATGAATGTA-3’ |
| BCAT2 | Human | 5’-TGCCTGGAGTGGTCAGACAGAG-3’ | 5’-GGTGCCCGAGCCAAAGACTTC-3’ |
| BCKDHA | Human | 5’-GCCGAAGGAGAAGGTGCTGAAG-3’ | 5’-TGCCGCTGAGACTCATAGAGGATG-3’ |
| BCKDHB | Human | 5’-GGTGGCTCATTTTACTTTCCAG-3’ | 5’-AGGATCTTTGGCCAATGAGTTA-3’ |
| Histidase | Human | 5’-GAAGAGGGTGCAGAAATCCA-3’ | 5’-TCCGACACCTCTCAGGACTT-3’ |
| SETD3 | Human | 5’-CAACCTGGAAGATGACCGCTGT-3’ | 5’-CACTGTGGATCACAAACTCTGCG-3’ |
| CNDP2 | Human | 5’-TAAGAAACTCGCAAAATGGGTG-3’ | 5’-CAACTTCCATCATCCTCCTGAT-3’ |
| GAPDH | Human | 5’-CAGGAGGCATTGCTGATGAT-3’ | 5’-GAAGGCTGGGGCTCATTT-3’ |

**Supplemental Table 5.** Mass spectrometry parameters for histidine and valine metabolites

| **Chemical** | **CAS number** | **MRM transition** | **DP (V)** | **CE (V)** |
| --- | --- | --- | --- | --- |
| **Metabolite of histidine** |  |  |  |  |
| 1-Methylhistidine | 332-80-9 | 170→124 | 40 | 20 |
| 3-Methylhistidine | 368-16-1 | 170→96 | 40 | 29 |
| Anserine | 584-85-0 | 241→109 | 80 | 30 |
| Carnosine | 305-84-0 | 227→110 | 40 | 25 |
| Ergothioneine | 497-30-3 | 230.3→127 | 40 | 24 |
| Homocarnosine | 584-85-0 | 240.9→95.2 | 30 | 30 |
| Trans-urocanate | 3465-72-3 | 139→93 | 24 | 24 |
| **Metabolite of valine** |  |  |  |  |
| 3-Methyl-2-Oxobutyrate | 3715-29-5 | 139→93 | 24 | 24 |

CE, collision energy; DP, declustering potential; MRM, multiple reaction monitoring; MS, mass spectrometry.

**Supplemental Table 6.** Gradient program for detection of histidine and valine metabolites

| **Total time (min)** | **Flow rate (μl/min)** | **A^a^ (v/v, %)** | **B^b^ (v/v, %)** |
| --- | --- | --- | --- |
| 0 | 300 | 95.0 | 5 |
| 0.50 | 300 | 95.0 | 5 |
| 3.00 | 300 | 5.0 | 95.0 |
| 3.50 | 300 | 5.0 | 95.0 |
| 4.00 | 300 | 95.0 | 5 |
| 6.50 | 300 | 95.0 | 5 |

^a^Solvent A, water containing 0.1% formic acid (v/v) and 0.05% trifluoroacetic acid; ^b^Solvent B, acetonitrile.

**Supplemental Table 7.** Plasma levels of the 20 amino acids in study participants.

| **AA**  **(μM)** | **CON**  **(n=30)** | **T2DM**  **(n=30)** | **DKD**  **(n=30)** | ***P1*** | ***P2*** | ***P3*** |
| --- | --- | --- | --- | --- | --- | --- |
| Gly | 194.8±96.1 (74.2-566.4) | 209.5±83.8 (40.8-361.0) | 215.7±86.5 (107.6-449.5) | 0.787 | 0.367 | 0.526 |
| Ala | 229.7±59.2 (116.2-339.8) | 271.6±83.3 (144.6-405.4) | 234.4±83.2 (127.8-453.5) | 0.062 | 0.811 | 0.036 |
| Arg | 203.8±51.5 (113.9-313.0) | 234.9±73.1 (116.2-412.5) | 211.6±75.3 (84.8-403.5) | 0.186 | 0.657 | 0.079 |
| Asn | 31.5±12.1 (9.9-59.3) | 38.6±10.4 (20.2-70.2) | 38.5±16.6 (11.8-78.6) | 0.980 | 0.045 | 0.043 |
| Asp | 39.0±16.0(10.7-72.2) | 51.4±20.9 (24.8-108.9) | 46.5±19.8 (13.9-92.3) | 0.317 | 0.133 | 0.013 |
| Cys | 264.3±66.6 (162.1-406.1) | 398.8±96.1 (246.6-564.8) | 291.7±72.9 (155.2-408.1) | <0.001 | 0.185 | <0.001 |
| Glu | 240.9±76.1 (113.7-468.7) | 376.8±219.5 (155.7-1247.8) | 312.1±89.8 (196.3-537.3) | 0.085 | 0.059 | <0.001 |
| His | 98.7±17.5 (67.6-135.3) | 102.7±19.5 (57.2-131.5) | 84.6±22.2 (46.7-123.2) | 0.001 | 0.007 | 0.446 |
| Iso | 65.3±12.2 (40.9-88.9) | 89.6±21.2 (51.7-146.5) | 80.1±27.7 (48.7-164.8) | 0.090 | 0.009 | <0.001 |
| Leu | 72.4±13.9 (45.4-101.3) | 94.9±22.3 (51.5-147.8) | 79.4±25.8 (45.4-150.8) | 0.006 | 0.205 | <0.001 |
| Lys | 109.3±14.1 (87.4-134.9) | 116.7±16.9 (82.0-157.4) | 114.6±20.7 (70.6-158.2) | 0.644 | 0.106 | 0.245 |
| Met | 49.6±9.8(35.7-70.7) | 55.6±14.7 (28.7-91.9) | 44.1±14.5 (20.7-87.1) | 0.001 | 0.104 | 0.081 |
| Orn | 48.7±21.7 (18.4-101.3) | 33.3±11.4 (19.2-65.2) | 53.6±24.1 (20.2-120.1) | <0.001 | 0.338 | 0.003 |
| Phe | 55.7±11.7 (34.9-86.1) | 57.2±11.6 (35.1-87.1) | 55.6±13.4 (33.9-93.6) | 0.618 | 0.990 | 0.627 |
| Pro | 110.7±32.8 (51.9-211.6) | 150.3±42.6 (86.9-229.4) | 141.6±47.8 (71.1-276.8) | 0.415 | 0.005 | <0.001 |
| Ser | 48.6±16.3 (28.4-88.1) | 47.3±17.0(22.1-84.9) | 44.7±16.9 (15.5-93.6) | 0.558 | 0.375 | 0.762 |
| Thr | 56.2±14.1 (37.9-100.9) | 60.7±16.1 (26.6-94.6) | 53.0±21.9 (29.4-135.6) | 0.096 | 0.088 | 0.328 |
| Trp | 41.7±6.4 (29.9-56.0) | 31.7±10.7(14.7-55.2) | \| 35.5±7.6(22.3-51.4) \| \| --- \| | 0.088 | <0.001 | 0.005 |
| Tyr | 44.3±8.8(29.3-63.2) | 50.7±11.4 (33.2-77.8) | 40.3±11.6 (20.4-77.9) | 0.000 | 0.145 | 0.024 |
| Val | 113.9±34.9 (67.6-203.7) | 122.3±20.1 (86.2-154.1) | 31.9±34.8(2.2-94.6) | <0.001 | <0.001 | 0.288 |

Data represent mean ± SD (range). P-values were determined by the one-way ANOVA test. P1: DKD vs T2DM group; *P2*: DKD vs CON group; *P3*: T2DM vs CON group; μM, micromole; CON, healthy controls; T2DM, type 2 diabetes mellitus; DKD, diabetic kidney disease; Gly, glycine; Ala, alanine; Arg, arginine; Asn, asparagine; Asp, aspartic acid; Cys, cysteine; Glu, glutamic acid; His, histidine; Iso, isoleucine; Leu, leucine; Lys, lysine; Met, methionine; Orn, ornithine; Phe, phenylalanine; Pro, proline; Ser, serine; Thr, threonine; Try, tryptophan; Tyr, tyrosine; Val, valine.

**Supplemental Table 8.** Relative quantification of histidine and valine metabolites in plasma of study participants

| **Chemical** | **CON**  **(n=20)** | **T2DM**  **(n=20)** | **DKD**  **(n=20)** | **P**  **value*** |
| --- | --- | --- | --- | --- |
| **Metabolite of Histidine** |  |  |  |  |
| 1-methylhistidine | 1.00±0.16 (0.61-1.35) | 0.77±0.21 (0.40-1.03) | 2.61±1.02 (1.20-4.23) | <0.001 |
| 3-methylhistidine | 1.00±0.57 (0.33-2.06) | 0.82±0.37 (0.34-1.60) | 3.63±1.66 (1.03-6.27) | <0.001 |
| Trans-urocanate | 1.00±0.42 (0.32-1.74) | 0.89±0.36 (0.42-1.61) | 0.83±0.53 (0.32-1.81) | 0.470 |
| Carnosine | 1.00±0.34 (0.44-1.47) | 0.61±0.24 (0.18-1.08) | 0.59±0.21 (0.32-1.16) | <0.001 |
| Homocarnosine | 1.00±0.33 (0.45-1.49) | 0.65±0.29 (0.33-1.55) | 0.90±0.33 (0.36-1.51) | 0.003 |
| Ergothioneine | 1.00±0.69 (0.22-2.44) | 0.71±0.30 (0.30-1.42) | 1.09±0.45 (0.43-1.90) | 0.058 |
| Anserine | 1.00±0.40 (0.17-2.13) | 2.75±1.28 (0.88-5.38) | 4.38±1.48 (1.96-7.00) | <0.001 |
| **Metabolite of Valine** |  |  |  |  |
| 3-methyl-2-oxobutyrate | 1.00±0.23 (0.54-1.42) | 2.20±0.77 (1.18-4.02) | 1.36±0.65 (0.31-3.12) | <0.001 |

The relative quantification of metabolites was performed by measuring the peak areas of the mass spectrum (MS) and scaling by the mean MS peak area in the control group.

Data represent mean ± SD (range).

**P* value determined using one-way analysis of variance among the 3 groups. CON, healthy controls; DKD, diabetic kidney disease; T2DM, type 2 diabetes mellitus.

**Supplemental Table 9.** Urine levels of the 20 amino acids in study participants.

| **AAs**  **(μM)** | **CON**  **(n=30)** | **T2DM**  **(n=30)** | **DKD**  **(n=30)** | ***P1*** | ***P2*** | ***P3*** |
| --- | --- | --- | --- | --- | --- | --- |
| Gly | 195.4±144.6(17.4-656.0) | 116.1±72.6(15.9-311.0) | 82.5±46.2(20.6-196.0) | 0.181 | 0.000 | 0.002 |
| Ala | 125.3±116.2(19.2-515.0) | 143.5±157.1(21.6-845.0) | 121.5±140.1(31.9-790.0) | 0.542 | 0.916 | 0.614 |
| Arg | 226.5±217.7(32.5-1075.0) | 242.2±148.1(39.2-750) | 169.9±125.4(18.9-426.5) | 0.100 | 0.197 | 0.718 |
| Asn | 40.9±33.0(3.03-161.0) | 44.8±54.7(5.0-262.0) | 32.9±33.7(1.5-154.0） | 0.274 | 0.461 | 0.719 |
| Asp | 668.6±374.6(153.0-1930) | 362.0±167.9(120.0-780.0) | 339.3±204.9(44.3-936.0) | 0.741 | 0.000 | 0.000 |
| Cys | 308.9±200.3(86.4-892.0) | 191.7±88.5(77.6-392.0) | 203.3±108.2(55.6-458.0) | 0.750 | 0.005 | 0.002 |
| Glu | 552.1±277.2(113.0-1140) | 375.3±203.3(108.0-1080.0) | 318.8±182.0(58.6-901.0) | 0.332 | 0.000 | 0.003 |
| His | 483.9±276.7(50.2-1200.0) | 271.7±164.1(41.7-826.0) | 159.3±174.5(26.0-829.0) | 0.042 | 0.000 | 0.000 |
| Iso | 8.5±14.8(0.7-54.8) | 18.5±13.2(1.5-49.6) | 8.5±9.5(0.4-33.2) | 0.003 | 0.999 | 0.003 |
| Leu | 13.1±18.3(2.2-86.4) | 22.4±15.9(2.4-68.6) | 12.9±12.8(2.3-54.0) | 0.023 | 0.972 | 0.025 |
| Lys | 119.5±65.6(14.1-323.0) | 85.7±100.2(9.2-430.0) | 73.7±64.2(8.8-311.0) | 0.557 | 0.026 | 0.098 |
| Met | 35.4±19.9(2.2-67.6) | 34.8±61.3(1.0-346.0) | 22.9±13.3(8.2-75.4) | 0.230 | 0.206 | 0.949 |
| Orn | 41.5±32.6(3.2-166.0) | 48.9±62.0(5.3-281.0) | 34.0±33.6(0.8-129.0) | 0.201 | 0.520 | 0.522 |
| Phe | 180.4±118.1(8.4-494.0) | 126.8±141.6(6.9-628) | 75.3±46.1(18.5-224.0) | 0.072 | 0.000 | 0.062 |
| Pro | 6.6±3.4(1.1-17.4) | 6.7±4.3(1.3-23.0) | 10.1±7.2(2.0-33.8) | 0.014 | 0.011 | 0.931 |
| Ser | 220.8±145.9(17.6-737.2) | 113.2±80.7(16.4-423.4) | 93.9±62.9(18.2-315.3) | 0.470 | 0.000 | 0.000 |
| Thr | 118.8±58.9(23.5-285.0) | 105.5±54.1(16.8-223.0) | 109.2±82.7(26.7-390.0) | 0.834 | 0.573 | 0.440 |
| Trp | 44.7±28.0(2.0-96.5) | 23.8±22.3(1.3-105.0) | 16.9±10.4(3.1-42.1) | 0.222 | 0.000 | 0.000 |
| Tyr | 13.3±10.9(0.1-38.9) | 3.2±2.1(0.1-7.8) | 5.2±4.6(0.1-25.4) | 0.252 | 0.000 | 0.000 |
| Val | 31.8±18.4(2.9-76.8) | 34.2±26.6(2.8-113.8) | 21.5±9.1(4.4-44.4) | 0.013 | 0.041 | 0.642 |

Data represent mean ± SD (range). P-values were determined by the ne-way ANOVA test. *P1*: DKD vs T2DM group; *P2*: DKD vs CON group; *P3*: T2DM vs CON group; μM, micromole; CON, healthy controls; T2DM, type 2 diabetes mellitus; DKD, diabetic kidney disease.

**Supplemental Table 10.** Saliva levels of the 20 amino acids in study participants.

| **AAs**  **(μM)** | **CON**  **(n=30)** | **T2DM**  **(n=30)** | **DKD**  **(n=30)** | ***P1*** | ***P2*** | ***P3*** |
| --- | --- | --- | --- | --- | --- | --- |
| Gly | 154.1±140.7(12.4-610.2) | 239.7±180.5(38.4-713.4) | 234.6±163.1(52.6-660.5) | 0.904 | 0.058 | 0.045 |
| Ala | 120.6±108.9(18.9-456.5) | 195.6±153.9(54.0-819.9) | 178.4±141.6(32.6-639.8) | 0.625 | 0.104 | 0.036 |
| Arg | 103.9±97.3(2.0-351.2) | 124.1±105.5(7.2-452.6) | 196.9±168.1(5.0-698.8) | 0.030 | 0.006 | 0.541 |
| Asn | 47.4±29.8(9.9-122.1) | 42.7±30.2(9.0-114.5) | 30.5±18.4(3.2-77.3) | 0.110 | 0.024 | 0.493 |
| Asp | 26.8±14.5(7.3-75.1) | 35.0±21.4(8.1-104.6) | 31.2±20.5(7.5-92.7) | 0.447 | 0.371 | 0.100 |
| Cys | 135.0±43.0(53.7-216.0) | 162.2±66.0(61.4-288.0) | 159.9±61.0(55.2-313.7) | 0.879 | 0.097 | 0.071 |
| Glu | 101.1±73.6(17.9-289.4) | 174.1±133.0(26.2-578.1) | 142.2±96.1(23.0-434.1) | 0.238 | 0.129 | 0.008 |
| His | 24.1±12.6(5.5-51.2) | 28.2±17.5(7.1-96.4) | 27.6±18.4(8.6-87.1) | 0.901 | 0.404 | 0.338 |
| Ile | 9.6±8.7(1.3-40.2) | 16.8±16.9(2.4-72.6) | 16.8±14.6(2.2-59.1) | 0.996 | 0.050 | 0.051 |
| Leu | 16.2±10.8(2.8-42.4) | 19.3±20.3(2.8-84.5) | 18.9±16.3(2.6-70.6) | 0.909 | 0.552 | 0.480 |
| Lys | 42.3±27.5(7.0-106.0) | 48.3±31.5(11.2-135.9) | 43.4±23.5(12.6-101.4) | 0.498 | 0.873 | 0.403 |
| Met | 9.2±13.9(0.4-70.9) | 19.6±34.9(1.6-194.9) | 16.6±18.1(1.2-78.3) | 0.634 | 0.239 | 0.100 |
| Orn | 51.9±31.6(10.4-142.2) | 51.3±39.2(7.9-182.8) | 46.6±32.8(2.7-157.8) | 0.598 | 0.550 | 0.943 |
| Phe | 26.3±15.0(5.7-69.2) | 45.5±38.9(10.4-185.9) | 38.0±25.3(7.7-103.7) | 0.306 | 0.109 | 0.010 |
| Pro | 27.3±21.6(3.0-81.9) | 45.4±36.6(7.8-172.6) | 34.5±27.9(8.4-126.6) | 0.155 | 0.347 | 0.019 |
| Ser | 14.9±13.8(1.9-51.5) | 24.3±21.6(4.0-101.6) | 19.6±17.8(4.5-77.0) | 0.314 | 0.309 | 0.045 |
| Thr | 17.9±15.4(3.5-63.0) | 25.9±25.3(4.3-138.0) | 21.6±16.5(5.1-64.5) | 0.398 | 0.474 | 0.120 |
| Trp | 11.5±10.4(1.5-43.1) | 27.2±33.3(3.7-147.4) | 18.8±11.7(3.3-41.3) | 0.132 | 0.186 | 0.005 |
| Tyr | 2.2±1.0(0.6-4.6) | 3.1±2.7(0.9-12.2) | 2.4±1.1(0.9-4.7) | 0.158 | 0.594 | 0.053 |
| Val | 13.3±12.0(1.7-50.5) | 27.1±28.6(4.0-131.0) | 22.8±17.5(2.9-80.9) | 0.420 | 0.077 | 0.011 |

Data represent mean ± SD (range). P-values were determined by the ne-way ANOVA test. *P1*: DKD vs T2DM group; *P2*: DKD vs CON group; *P3*: T2DM vs CON group; μM, micromole; CON, healthy controls; T2DM, type 2 diabetes mellitus; DKD, diabetic kidney disease.

**Supplemental Table 11.** Correlation analysis between plasmatic and urinary or salivary amino acids

| **AAs(μM)** | | **Plasma concentration** | **Urine concentration** | **Salivary concentration** | **r** | ***P*** |
| --- | --- | --- | --- | --- | --- | --- |
| His | 84.6±22.2(46.7-123.2) | | 159.3±174.5(26.0-829.0) | / | 0. 538 | 0.000 |
| Pro | 141.6±47.8 (71.1-276.8) | | 10.1±7.2(2.0-33.8) | / | 0.141 | 0.186 |
| Val | 73.4±18.2(36.4-109.9) | | 21.5±9.1(4.4-44.4) | / | 0.685 | 0.000 |
| Arg | 211.6±75.3(84.8-403.5) | | / | 196.9±168.1(5.0-698.8) | 0.165 | 0.121 |

r: Pearson’s Correlations Coefficient.

**Supplemental Table 12.** Plasma, urine and saliva Logistics regression model parameters

|  | B | S.E. | Wals | df | sig | Exp（B） |
| --- | --- | --- | --- | --- | --- | --- |
| Plasma His | -0.026 | 0.025 | 1.008 | 1 | 0.315 | 0.975 |
| Plasma Val | -0.099 | 0.029 | 11.929 | 1 | 0.001 | 0.906 |
| Urine His | -0.004 | 0.002 | 2.929 | 1 | 0.087 | 0.996 |
| Urine Val | 0.002 | 0.046 | 0.002 | 1 | 0.967 | 1.002 |
| Urine Pro | 0.064 | 0.110 | 0.341 | 1 | 0.559 | 1.066 |
| Saliva Arg | 0.006 | 0.004 | 3.003 | 1 | 0.083 | 1.006 |
| Constant | 10.673 | 3.551 | 9.032 | 1 | 0.003 | 43157.133 |

## Supplementary Figures

| 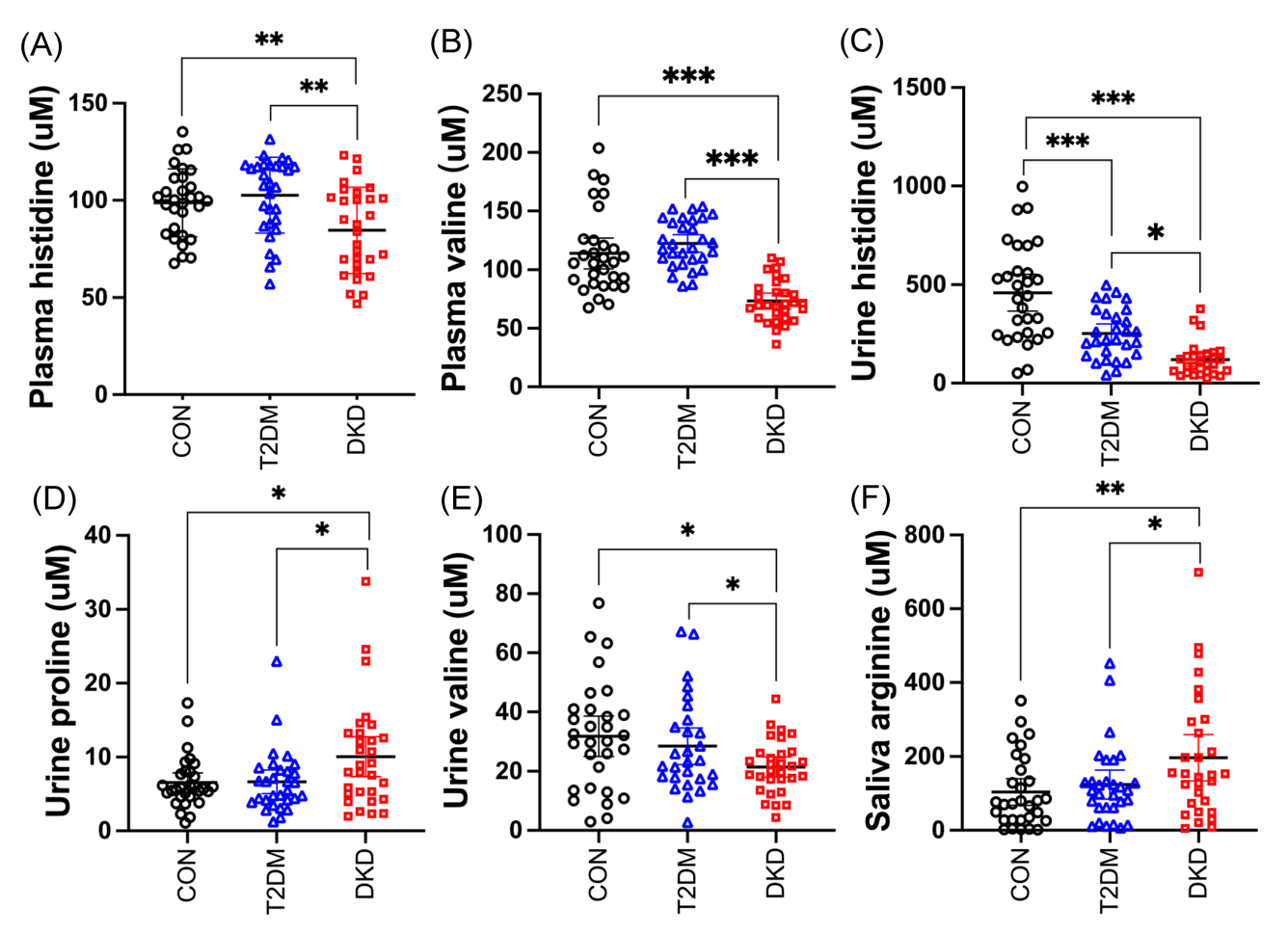 |
| --- |
| **Supplemental Figure 1. Plasma levels of histidine and valine; urine levels of histidine, proline and valine; and, saliva levels of arginine have an excellent predictive value for separating patients with DKD from those with T2DM, and healthy controls.** Plasma levels of (A) histidine, (B) valine; urine levels of histidine (C), proline (D), and valine (E); saliva levels of arginine (F) in the CON, T2DM, and DKD groups, respectively. **P* < 0.05, ***P* < 0.01, ****P* < 0.001. CON, healthy control; DKD, diabetic kidney disease; T2DM, type 2 diabetes mellitus. |

| 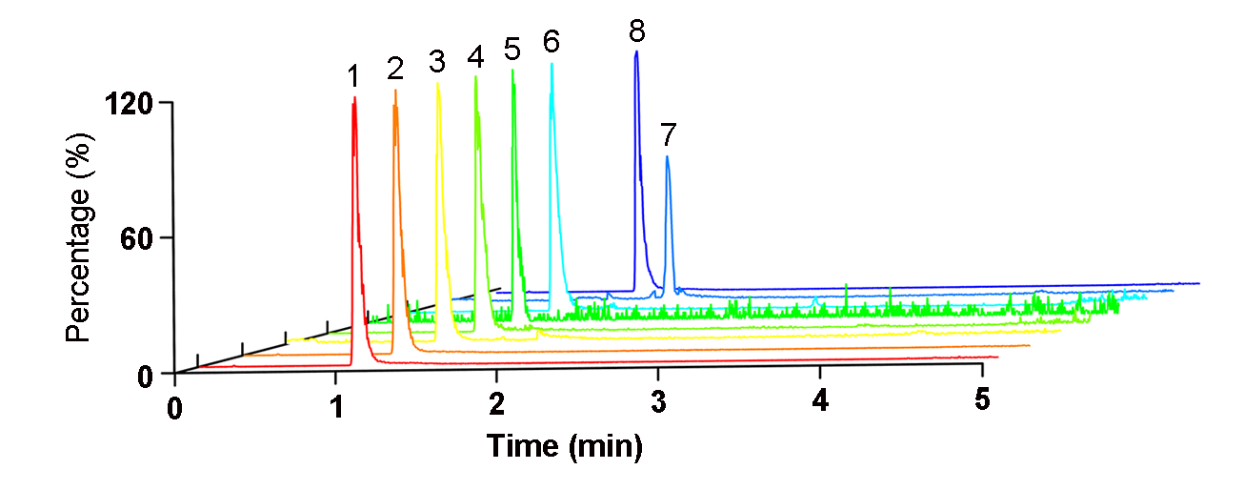 |
| --- |
| **Supplemental Figure 2. Chromatogram of histidine and valine metabolites.**  1, 1-methylhistidine; 2, 3-methylhistidine; 3, 3-methyl-2-oxobutyrate; 4, carnosine; 5, ergothioneine; 6, homocarnosine; 7, trans-urocanate; 8, anserine. |

**References**

1. Microvascular Complications and Foot Care: Standards of Medical Care in Diabetes—2019. *Diabetes Care,* 42**:** S124-S138, 2019 10.2337/dc19-S011

2. Classification and Diagnosis of Diabetes: Standards of Medical Care in Diabetes—2019. *Diabetes Care,* 42**:** S13-S28, 2018 10.2337/dc19-S002
